# Short-Term Celecoxib Promotes Bone Formation without Compromising Cefazolin Efficacy in an Early Orthopaedic Device-Related Infection: Evidence from a Rat Model

**DOI:** 10.3390/antibiotics13080715

**Published:** 2024-07-30

**Authors:** Vuyisa Siphelele Mdingi, Lena Gens, Karen Mys, Peter Varga, Stephan Zeiter, Leonard Charles Marais, Robert Geoffrey Richards, Fintan Thomas Moriarty, Marco Chittò

**Affiliations:** 1 AO Research Institute Davos, 7270 Davos, Switzerland; mdingiv@ukzn.ac.za (V.S.M.);; 2 Department of Orthopaedic Surgery, School of Clinical Medicine, University of KwaZulu Natal, Durban 4041, South Africa

**Keywords:** non-steroidal anti-inflammatory drugs (NSAIDs), cyclooxygenase enzymes (COX-1 or COX-2), bone healing, orthopaedic device-related infection (ODRI)

## Abstract

Non-steroidal anti-inflammatory drugs (NSAIDs) are crucial components of multimodal analgesia for musculoskeletal injuries, targeting cyclooxygenase (COX) enzymes (COX-1 and/or COX-2 isoenzymes). Concerns exist regarding their potential interference with bone healing and orthopaedic device-related infections (ODRI), where data are limited. This study aimed to investigate whether the COX-selectivity of NSAIDs interfered with antibiotic efficacy and bone changes in the setting of an ODRI. In vitro testing demonstrated that combining celecoxib (a COX-2 inhibitor) with cefazolin significantly enhanced antibacterial efficacy compared to cefazolin alone (*p* < 0.0001). In vivo experiments were performed using *Staphylococcus epidermidis* in the rat proximal tibia of an ODRI model. Long and short durations of celecoxib treatment in combination with antibiotics were compared to a control group receiving an antibiotic only. The long celecoxib treatment group showed impaired infection clearance, while the short celecoxib treatment showed increased bone formation (day 6, *p* < 0.0001), lower bone resorption (day 6, *p* < 0.0001), and lower osteolysis (day 6, BV/TV: *p* < 0.0001; BIC: *p* = 0.0005) compared to the control group, without impairing antibiotic efficacy (*p* > 0.9999). Given the use of NSAIDs as part of multimodal analgesia, and considering these findings, short-term use of COX-2 selective NSAIDs like celecoxib not only aids pain management but also promotes favorable bone changes during ODRI.

## 1. Introduction

Non-steroidal anti-inflammatory drugs (NSAIDs) are among the most prescribed drugs worldwide [1,2]. In the orthopaedic setting, they are used for their analgesic and anti-inflammatory properties as part of the multimodal management of pain while minimizing opioid-related adverse effects [3].

NSAIDs can be classified into different classes based on their chemical structures and work by inhibiting cyclooxygenase (COX), the rate-limiting enzyme for prostaglandins (PG) synthesis, which is important in mediating pain and inflammation responses. NSAIDs work by inhibiting COX-1 and COX-2, which are the two major isoforms. Non-selective NSAIDs, such as aspirin, ibuprofen, and naproxen, inhibit both COX-1 and COX 2 isoforms, providing effective relief from pain, inflammation, and fever. Despite the positive effects, their potential complications include gastrointestinal irritation and increased bleeding risk. In contrast, selective COX-2 inhibitors, such as celecoxib, specifically target the COX-2 isoform, aiming to reduce pain and inflammation with a lower risk of gastrointestinal complications when compared to non-selective NSAIDs. While celecoxib is the only Food and Drug Administration (FDA)-approved COX-2 inhibitor, other drugs have been discontinued due to concerns over adverse cardiovascular effects [4].

One important aspect to consider when using NSAIDs in fracture patients is the potential influence they may have on the bone healing process. The proposed mechanism for the interference in bone healing is due to the inhibition of prostaglandin synthesis, particularly PGE2. PGE2 interacts with two important receptors, EP2R and EP4R, which stimulate bone formation and bone resorption, respectively [5,6,7].

A recent meta-analysis by Al Farii et al. and Wheatley et al. independently demonstrated an elevated risk of non-union in patients receiving NSAIDs, especially when the duration of the treatment exceeded four weeks [8,9]. However, Marquez-Lara et al. could not make any recommendations regarding the safety of NSAID use as there was a poor quality of studies that showed the negative effect of NSAIDs compared with those that concluded that NSAIDs were safe [10]. A few studies investigated the bone protective effects in postmenopausal patients. It has been shown that regular use of aspirin only, or combined with a relative COX-2 selective NSAID, is associated with a higher bone mineral density (BMD) when compared to the nonuser [11,12]. This has been further supported with lower serum alkaline phosphatase concentrations [13]. While the increase in BMD has been demonstrated, there is still conflicting evidence regarding the effect on reducing the fracture risk. Bauer et al. found no significant protective effect on the risk of fractures, while Mao et al. found a combination of aspirin with clopidogrel for three or more years effective in minimizing the risk of hip fractures [14,15].

Regarding increased bone infection risk in patients, Jeffcoach et al. showed that patients with long bone fractures who received NSAIDs post-operatively were twice as likely to suffer complications such as non-union, malunion, or infection [16].

While guidelines exist for the use of NSAIDs in acute trauma, uncertainty regarding their use in bone and joint infections is a concrete concern [17]. Only a few studies have investigated whether NSAIDs impair antibiotic efficacy when used concurrently in the treatment of bone infections. In one of those studies, where a rat model was used, it was shown that carprofen (another selective COX-2 inhibitor) negatively affected antibiotic efficacy in clearing *Staphylococcus epidermidis* infection [18]. This study also demonstrated that the concurrent administration of carprofen and antibiotics resulted in a reduction in *S. epidermidis*-induced osteolysis and significantly suppressed reparative bone-forming processes. Nevertheless, the precise mechanism as well as the length of the treatment underlying the interaction between NSAIDs and antibiotics remains unclear. It appears that NSAID administration may potentially enhance bacterial survival in the presence of antibiotics. Further animal studies have found these effects to be either dose- or duration-dependent [19,20]. The effect seems to be greater with COX-2 selective inhibitors compared to non-specific NSAIDs [21].

As shown in a previous study, the concurrent administration of carprofen and antibiotics led to an inadequate clearance of infections and an overall diminished occurrence of osteolysis. In this study, we investigated the effect of different durations of celecoxib treatment on the efficacy of antibiotics in a rat model. Consequently, we hypothesized that pre-treatment with celecoxib could serve to safeguard bone integrity against osteolysis, followed by independent administration of antibiotics to mitigate and resolve the infection. We assessed the impact of these different durations on bone changes and reduction in bacterial load. Additionally, we sought to ascertain whether the observed effects were specific to COX inhibition and subsequently extended our investigation to encompass other classes of NSAIDs, including acetylsalicylic acid (ASA) and ibuprofen.

## 2. Results

### 2.1. Antibacterial Effect of NSAIDs in Combination with Antibiotics In Vitro

**Figure 1:**
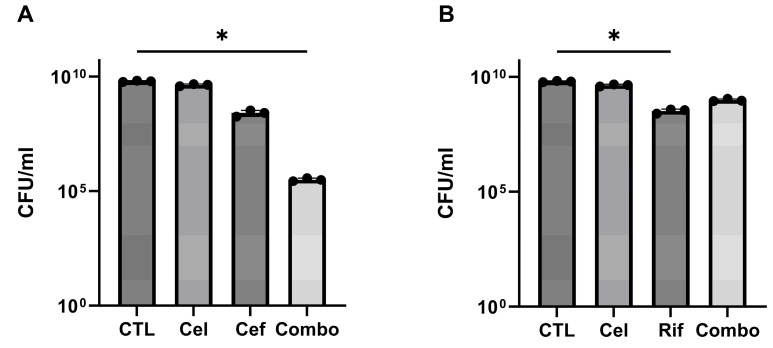
In vitro antimicrobial activity of (**A**) cefazolin 0.3 µg/mL (1×MIC) and celecoxib 250 µg/mL alone or in combination and (**B**) rifampicin 0.2 µg/mL (1×MIC) and celecoxib 250 µg/mL alone or in combination. Each bar plot represents the average of three independent experiments with three replicates per experiment. A one-way ANOVA with Tukey’s post hoc test was performed to determine significant differences between the groups. Standard deviation and significance level are shown. * *p* < 0.05.

The in vitro assessment against planktonic *S. epidermidis* demonstrated, as illustrated in Figure 1A, that combined exposure to 250 µg/mL celecoxib and 0.3 µg/mL cefazolin resulted in a higher antibacterial efficacy when compared with cefazolin alone (*p* = 0.0127). Similarly, combining cefazolin with ASA and/or ibuprofen also demonstrated a beneficial effect in reducing the bacterial load (Supplementary Figure S1A,B). However, combining celecoxib with rifampicin under the same conditions did not result in a significant improvement when compared to the action of the antibiotic alone (*p* = 0.0134) (Figure 1B). Similarly, no beneficial effect was observed with the combination of ASA and/or ibuprofen with rifampicin (Supplementary Figure S1C,D).

### 2.2. Animal Welfare

All animals recovered well from surgery and anesthesia. A total of six animals had to be excluded from the study: three intraoperatively due to a misplaced screw, two due to deep wound opening—a predefined exclusion criterion (day 6 and 7)—and one due to severe lameness (day 9). All these excluded animals were replaced by reserve rats. Mean weight loss following surgery was 6.6% of initial body weight at seven days and no animal was excluded due to excessive weight loss. Additionally, the surgical wounds of five animals opened 4–6 days post-op, but as only the skin was affected, no secretion occurred, and the wound healed by secondary wound healing, these animals were retained in the study.

### 2.3. The Impact of NSAIDs on the Clearance of Infection

**Figure 2:**
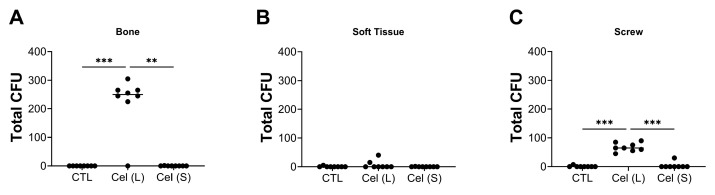
Quantitative bacteriological assessments at euthanasia (day 28) of the control (CTL), long (L), and short (S) celecoxib (Cel)-treated groups from the different locations: (**A**) bone, (**B**) soft tissue, and (**C**) screw. Data shown are the total CFU counts from each animal with mean and standard deviation. A Kruskal–Wallis test with Dunnett’s post hoc test for multiple comparisons was performed to compare differences in CFU counts between groups. The significance levels are shown. ** *p* < 0.01, *** *p* < 0.001.

The tissues and screws collected from each animal after the study were processed as described in Section 4 for quantitative bacterial quantification. Control animals, which were infected and subjected to systemic antibiotic treatment, showed an absence of bacterial growth both in soft tissue and screws, with only a limited number of colonies recoverable from the bone samples. The simultaneous administration of a short-term celecoxib regimen with antibiotics did not increase the bacterial load compared to the control cohort. However, the concurrent administration of long-term celecoxib with antibiotics demonstrated a significant reduction in the treatment efficacy, with an increase in bacterial load at the time of euthanasia (bone: comparison Cel (L) with Cel (S) *p* = 0.0026; screw: comparison Cel (L) with Cel (S) *p* = 0.0005) (Figure 2). Total CFU quantification including all the NSAIDs that were used in this study are shown in Supplementary Figure S2.

### 2.4. Bone Changes in the Vicinity of the Implant

**Figure 3:**
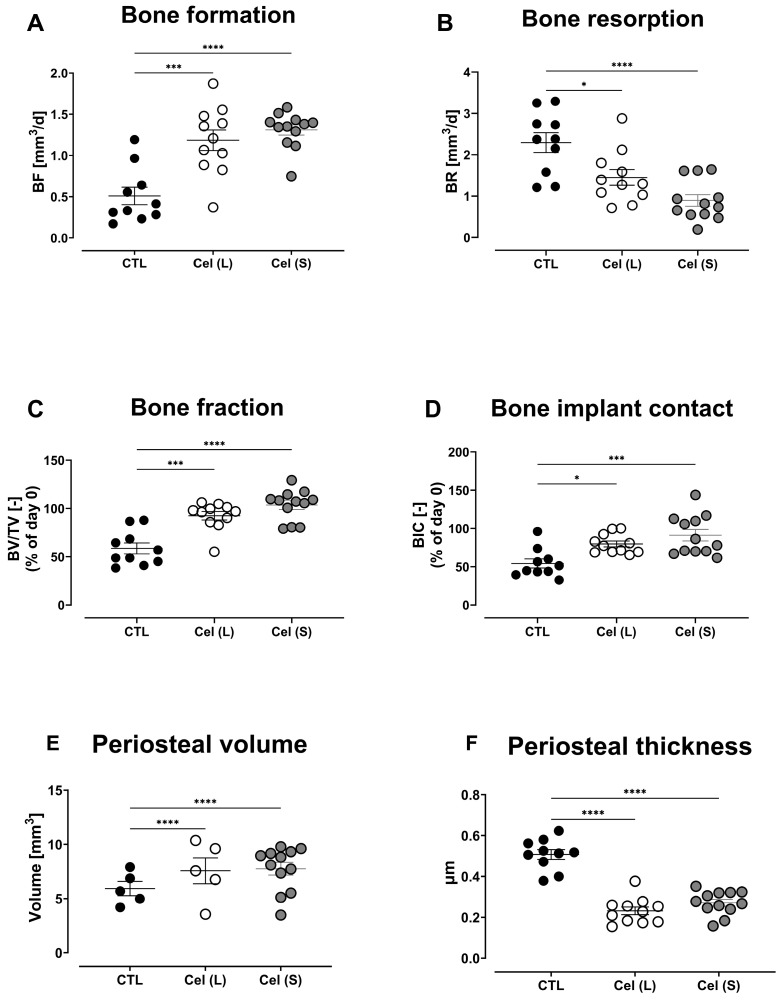
Bone changes in the control (CTL), long-term celecoxib (Cel(L)), and short-term celecoxib (Cel(S)) treatment groups of an *S. epidermidis* infection. Differences in day 6 bone formation (**A**), bone resorption (**B**), bone fraction (**C**), and bone–implant contact (**D**). Day 14 periosteal reaction is shown as the periosteal volume (**E**) and periosteal thickness (**F**). Data shown are the mean ± SEM. A two-way ANOVA with Dunnett’s post hoc test was performed to determine significant differences between treatment groups and the control group. * *p* < 0.05, *** *p* < 0.001, **** *p* < 0.0001.

The effect of antibiotic and NSAID combination therapy on the bone turnover, osseointegration of the implant, and the periosteal reaction was assessed by µCT analysis over different time points. There was an increasing trend in bone formation in the control group with a peak in bone formation observed at day 20 (mean ± SEM = 2.554 ± 0.2918 mm^3^/d), which then reduced by day 28 (mean ± SEM = 1.541 ± 0.2112 mm^3^/d). Similarly, a significant increase in bone formation was observed in the long celecoxib treatment group and this was significantly higher compared to the control group (day 6, *p* = 0.0002). This increase was also observed in the short celecoxib treatment group (day 6, *p* < 0.0001) (Figure 3A). Bone resorption in the control group peaked on day 6 (mean ± SEM = 2.293 ± 0.2393 mm^3^/d) and reached its lowest value on day 20 (mean ± SEM = 0.5457 ± 0.1273 mm^3^/d). The long celecoxib treatment group had significantly lower bone resorption compared to the control group (day 6, *p* = 0.0118), and a similar difference was also observed in the short celecoxib treatment group (day 6, *p* < 0.0001) (Figure 3B).

There was increased osteolytic activity in the control group from day 0 to day 6 demonstrated by a decrease in bone fraction (bone volume (BV)/total volume (TV)) and bone–implant contact (BIC). The long celecoxib treatment group showed significantly lower osteolytic activity compared to the control group on day 6 (BV/TV: *p* = 0.0001; BIC: *p* = 0.0183). Similar results were observed in the short celecoxib treatment group (BV/TV: *p* < 0.0001; BIC: *p* = 0.0005) (Figure 3C,D).

The periosteal reaction was increased in the control group, peaking on day 14 (mean periosteal volume ± SEM = 15.19 ± 1.25 mm^3^). The periosteal reaction was significantly lower in the long celecoxib treatment group compared to the control group (periosteal volume: day 14, *p* < 0.0001; day 20, *p* < 0.0001). A similar significant difference was observed in the short celecoxib treatment group (day 14, *p* < 0.0001; day 20, *p* < 0.0001; day 28, *p* = 0.0008) (Figure 3E,F).

The bone change data for the other NSAID groups and the comparison between long and short celecoxib treatment compared to the control group are shown, respectively, in Supplementary Figures S3A–D and S4A–C. In summary, there was no significant difference in bone formation and bone resorption in the ASA group compared to the control group. In the ibuprofen group, there was no difference in bone formation compared to controls but there was significantly lower bone resorption at day 6 (*p* = 0.0160). The ASA group showed no difference in osteolytic activity compared to controls, while we observed lower osteolytic activity in the ibuprofen group (day 6 BV/TV: *p* = 0.0282). While there was no difference in the periosteal reaction in the ASA group compared to the controls, there was a significantly lower periosteal reaction in the ibuprofen treatment group (periosteal volume: day 14, *p* = 0.0393; day 20, *p* = 0.0271) (Supplementary Figure S3E,F).

### 2.5. Histomorphometric Analysis

**Figure 4:**
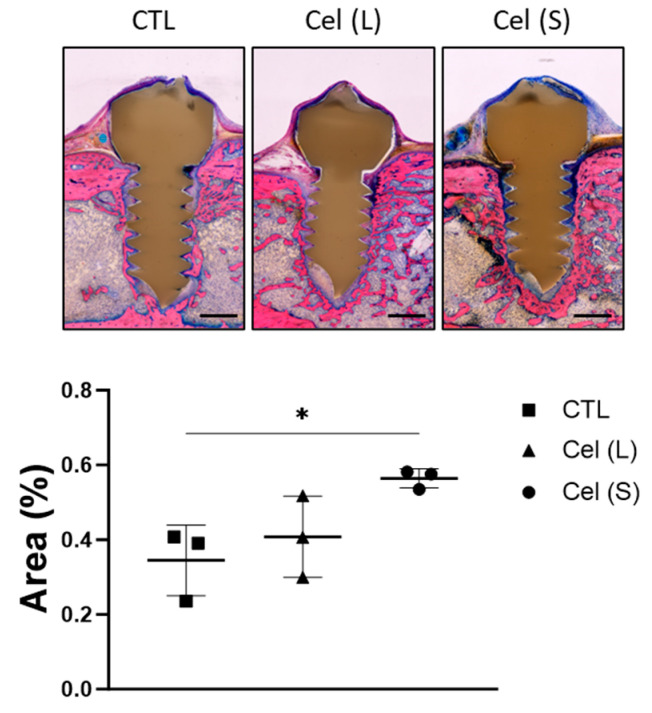
Histological evaluations from control (CTL), long-term celecoxib (Cel (L)), and short-term celecoxib (Cel (S)) treatment groups. Representative images of the screw with the bone content of the selected ROI used for quantification are shown. The 50–60 μm thick sections, embedded in methyl methacrylate (MMA), were cut in the frontal plane through the center of the PEEK implant. Magnification objective 4×, scale bar of 500 µm. The sections were stained with Giemsa–Eosin, and the proportion of trabecular bone present in a defined ROI was quantified using the ImageJ software. A one-way ANOVA with Dunnett’s post hoc test was performed to determine significant differences between treatment groups and the control group * *p* < 0.05.

The quantity of bone present in the region of interest (ROI) was evaluated. Animals that received short-term celecoxib treatment displayed a statistically significant increase in bone formation (*p* = 0.0338) compared to the results of the long-term celecoxib treatment and the control group (Figure 4).

### 2.6. Blood Cytokine Analysis

Blood samples collected at various time points during the study were processed to assess cytokine and chemokine secretion using a multiplex immunoassay (Meso Scale Discovery, Rockville, MD, USA). The analysis indicated no statistically significant differences in the levels of either pro-inflammatory or anti-inflammatory cytokines among the short-term and long-term celecoxib treatment groups, compared to the control group, as depicted in Supplementary Figure S5. Despite the previously observed antagonistic effects of the celecoxib–antibiotic combination treatment among the other treatment groups, there were no statistically significant disparities at the cytokine level. These results remained consistent (Supplementary Figure S6).

## 3. Discussion

NSAIDs, in combination with other analgesic agents, constitute an integral component of multimodal pain management for fracture patients. In the context of patients afflicted with musculoskeletal infections, NSAIDs are frequently administered concomitantly with antibiotics. Following the findings from Burch et al., the combination of an NSAID with antibiotics resulted in a deleterious effect in terms of infection resolution. Our study was designed to address a critical gap in the existing literature, with a specific focus on the COX specificity in the assessment of the reparative responses of bone tissue and the efficacy of antibiotics in the clearance of *S. epidermidis* infections.

Our in vitro investigation revealed that the efficacy of celecoxib is enhanced when combined with cefazolin but diminished when paired with rifampicin. In addition to its antimicrobial properties, celecoxib has been demonstrated to inhibit multidrug efflux pumps in *Mycobacterium smegmatis* and *S. aureus*, thereby enhancing bacterial susceptibility to a spectrum of antibiotics, including ampicillin, kanamycin, ciprofloxacin, and chloramphenicol [22]. Nonetheless, the precise antibacterial mechanism of action of celecoxib and its prospective clinical utility remains unexplored. Further in vivo investigations revealed that prolonged celecoxib administration in conjunction with antibiotics had a detrimental impact on the resolution of the infection while short-term celecoxib treatment did not appear to interfere with the efficacy of antibiotics in resolving the infection. On the other hand, ASA and ibuprofen did not interfere with antibiotics in clearing the infection in all tissues, including soft tissue, screws, and bones.

The celecoxib combination treatment exhibited a notable reduction in periosteal volume and periosteal thickness in comparison to the control group, as well as the ibuprofen- and aspirin-treated groups. The periosteal thickness observed in the control group is most likely derived from the periosteum’s response to the inflammatory and infectious processes within the bone. Several studies support the hypothesis that the presence of immune cells and inflammatory mediators in the affected site stimulates the periosteum, particularly the inner cambium layer, to produce new bone as a defense mechanism [23,24,25].

The reductions observed were significant at days 14 and 20 for both periosteal volume (*p* < 0.0001) and periosteal thickness (*p* < 0.0001 and *p* = 0.0003). Additionally, our analysis indicated that celecoxib displayed higher bone formation and reduced bone resorption values at the initial infection stages on day 6 (*p* < 0.05). The results suggest that celecoxib, specifically in the early stages of *S. epidermidis* osteomyelitis, may be a promising therapeutic option for preventing bone damage and promoting bone regeneration. It is important to notice that these findings align with prior studies conducted by Burch et al., which demonstrated similar effects on periosteal reaction, bone formation, and bone resorption when utilizing an alternative COX-2 inhibitor [18]. With respect to bone turnover, our study results indicate that COX-2 selective inhibitors, particularly celecoxib, exhibit the capacity to enhance bone formation and mitigate bone resorption during both short-term and long-term treatment, as opposed to the control group and other NSAID treatment cohorts. Notably, celecoxib treatment exerts a favorable influence on bone healing, particularly during the early stages of *S. epidermidis* osteomyelitis. Specifically, our observations reveal that celecoxib effectively restrains bone resorption while concurrently promoting bone formation, substantiated by higher BV/TV and BIC values in comparison to the control group and the other NSAID treatment groups. Interestingly, we found a reduced periosteal reaction with short-term celecoxib treatment, suggesting that the bone formation is mainly due to the endosteal blood supply. Those findings also highlight the importance of considering the timing and duration of NSAID administration when developing treatment plans for patients with bone infections. Ibuprofen has been shown to be a safe analgesic option in healing fractures. In a randomized clinical trial, Aliuskevicius et al.’s study showed no difference in bone mineral density, histomorphometric estimations, and changes in biomarkers between placebo and ibuprofen treatment groups in patients with Colles’ fractures [26,27]. In a rat experimental study that compared the biomechanical strength of tibia fracture healing between animals that received ibuprofen and celecoxib, Handoko et al. showed that ibuprofen had a lower bone healing suppression effect than celecoxib in bending and torsion tests [28]. While the effect of ibuprofen use during bone infection is not well known, the literature investigating its use has shown that it has no negative effect on wound healing [29]. It has also shown a trend to hasten the resolution of inflammation [30]. The underlying mechanism which celecoxib protects against bone damage caused by *S. epidermidis* osteomyelitis remains unknown. Most of the literature around COX-2 inhibitors shows that they interfere with secondary bone healing. Welting et al. demonstrated that COX-2 inhibition leads to reduced levels of bone-morphogenic protein-2 (BMP-2). This led to impaired chondrocyte hypertrophic differentiation [31]. One article showed positive effects of celecoxib use. Kasukawa et al. found that celecoxib treatment decreased serum C-telopeptide levels (a marker of bone resorption) in ovariectomized mice. The same study showed that celecoxib did not affect serum osteocalcin levels (a marker of bone formation). More research is needed to investigate the exact molecular and cellular pathways that are involved in the protective effects of celecoxib on bone health during an infection. Understanding these mechanisms could ultimately lead to the development of more targeted and effective treatments for osteomyelitis and other bone-related diseases.

In the present study, plasma cytokine data analysis did not reveal a systemic pro-inflammatory response caused by *S. epidermidis* during the 28-day study period. This indicates that the bacterial infection did not trigger a significant immune response in the bloodstream and suggests that the host response to the infection was primarily localized to the site of infection. This finding is interesting because it suggests that the bacteria may be able to evade detection by the immune system or that the immune response is not strong enough to elicit a systemic inflammatory response. On the other side, *S. epidermidis* lacks virulence factors that are normally expressed by the most virulent strain *S. aureus*, and those virulence factors are strong stimulators of inflammatory responses [32,33].

Possible areas of future research include the investigation of large animal studies as well as fracture and fixation models. The fracture models should include simple and comminated fractures, fixed with either plate and screws or an intramedullary nail. This would enable us to recreate the clinical scenario where we could test NSAID use in expected primary and secondary bone healing, distinct from the current paradigm, which primarily centers on the assessment of implant integration. Additionally, an intriguing aspect deserving a closer examination is related to the localized administration of NSAIDs. This approach requires a thorough investigation to determine its potential advantages, especially in reducing the systemic side effects commonly associated with conventional systemic NSAID administration. The evaluation of localized NSAID delivery represents a pivotal area that requires deeper investigation, aiming to enhance therapeutic strategies in the current orthopaedic settings. Local delivery of celecoxib is well-described in ophthalmology experimental research. A study by Ayalasomayajula et al. concluded that the retinal availability of celecoxib is 54-fold higher following subconjunctival (local) administration compared to intraperitoneal (systemic) administration [34]. In the orthopaedic literature, NSAIDs administered locally in and around the joint reduce post-operative pain scores and opioid consumption in patients undergoing arthroplasty [30]. Regarding the safety and efficacy of intra-articular (IA) NSAID injections in osteoarthritis (OA), Selig et al. found that IA NSAIDs had similar efficacy to oral NSAIDs in treating OA-related pain in humans [35]. Future studies should evaluate not only the efficacy of local NSAID administration but also the effects on bone responses following a fracture. The risk of infection with local administration in traumatized tissues is unknown and should also be explored.

## 4. Materials and Methods

### 4.1. In Vitro Study of NSAID and Antibiotic Combination Therapy

*Staphylococcus epidermidis* (strain 103.1; a clinical isolate from a patient with a chronic orthopaedic device-related infection and available from the Culture Collection of Switzerland, strain number CCOS 1152) was recovered from frozen stocks. Briefly, the bacterial strain was cultured in tryptic soy broth (TSB) liquid medium and incubated under shaking conditions overnight at 37 °C. On the day of the experiment, a subculture (dilution 1:200) was prepared and subsequently adjusted to a starting optical density of OD600 0.01 to target a concentration of approximately 10^6^ colony-forming units per milliliter (CFU/mL). Two antibiotics, cefazolin (Sandoz Pharmaceuticals AG, Rotkreuz, Switzerland) and rifampicin (Carl Roth GmbH, Karlsruhe, Germany), and three NSAIDs, ASA, ibuprofen, and celecoxib (Mepha Pharma AG, Basel, Switzerland), were tested. TSB, phosphate-buffered saline (PBS) tablets, and dimethyl sulfoxide (DMSO) were purchased from Sigma-Aldrich. NSAIDs were reconstituted by dissolving NSAID powder in pure DMSO, further diluted in 70% ethanol, and into sterile PBS to the target concentration. Antibiotic stocks were made by dissolving the antibiotic powder in PBS to the target concentration. The final DMSO concentration present after the dilution steps was approximately 10%. To assess the synergistic or antagonistic effects of combination treatment, a fixed concentration of the NSAIDs (250 µg/mL) was combined with a fixed concentration of either cefazolin (0.3 µg/mL) or rifampicin (0.2 µg/mL). The selected antibiotic concentrations were determined during preliminary testing for the minimum inhibitory concentration (MIC) defined as the lowest concentration that inhibits visible growth of an organism following overnight incubation [36].

### 4.2. In Vivo Study of NSAID and Antibiotic Combination Therapy

Consent to perform this study was granted by the ethical committee of the Canton of Grisons in Switzerland (approval numbers: 02/2022, 14E/2022, and 20/2022) and performed according to the Swiss Animal Protection Law in an AAALAC International-accredited facility. Our previously reported model of ODRI in the rat proximal tibia was used in this study, utilizing custom polyetheretherketone (PEEK) screws [37,38]. A total of 63 rats were included in this study (12 rats per group for the ASA, ibuprofen, short-term celecoxib, and control groups, and 15 rats for the long-term celecoxib group). An overview of the grouping and treatment duration can be seen in Supplementary Table S1. The sample size was based on previous studies using this model [18]. Groups were assigned randomly via drawing before surgery. PEEK screws pre-colonized with *S. epidermidis* were implanted into the right tibia, as described below. The inoculum (1 × 10^6^ CFU) was sufficient to guarantee infection in all animals based on experience from previous study [18]. Post-implantation, tibiae were monitored with in vivo micro-CT at four time points over a 28-day period during which they received different NSAIDs (COX-1, COX-2, non-selective) or the vehicle substance only, according to their group. In addition, a fifth group was included, which received a COX-2 inhibitor for only the first seven days after surgery. All analyses were performed in a blinded manner, and group assignment was not announced until data collection was completed.

### 4.3. Implant Design and Manufacturing

Custom-made screws (5 mm length, 1.5 mm diameter) were machined from medical-grade PEEK containing 20% (*w/w*) barium sulphate (material supplied by Invibio Biomaterials Ltd., Lancashire, UK) by RISystem AG, Davos, Switzerland.

### 4.4. Bacterial Inoculum Preparation

The bacterial inoculum was introduced to the rats on pre-contaminated PEEK screws prepared immediately before each surgery. Briefly, on the day of the surgery, *S. epidermidis* 103.1 overnight culture was centrifuged (2500× *g* for 10 min), washed twice in PBS, and adjusted to an optical density of OD600 0.50 (±0.01). The threaded portion of the screw was submerged and incubated statically at room temperature for 30 min. Similarly, test screws were inoculated in parallel within each series of experiments. A quantitative assessment of *S. epidermidis* 103.1 adhesion to the test screw was performed by sonication in PBS (3 min) followed by serial dilution, plating on 5% horse blood agar (Oxoid) incubated overnight at 37 °C. The target inoculum for each screw was set to 1 × 10^6^ CFU/screw (range: 0.9–2 × 10^6^ CFU/screw). The inoculum target concentration was chosen following a prior study in which it was empirically established that the selected concentration was sufficiently elevated to induce an infection yet maintained at a level that precluded mortality in the experimental animals [18]. All screws were implanted within 3 h of preparation.

### 4.5. Animal Welfare, Observation, and Euthanasia

Skeletally mature, 19 to 22 weeks old, female, specific pathogen-free (SPF) Wistar rats purchased from Charles River (Erkrath, Germany) were used in this study. Inclusion was granted after a clinical health examination by a veterinarian. They were housed in groups of 3, in individually ventilated or open cages until the start of the study, and in open cages during the study period with a 12-h light/dark cycle. Water and food (Kliba Nafag, Provimini Kliba SA, 3436 EXS12 S/R Entretien Extrude, Kaiseraugst, Switzerland, Complete food for mice and rats) were available ad libitum. During the acclimatization period, which lasted 48–85 days, the rats were accustomed to diluted condensed milk (1:10 condensed milk–tap water), which they learned to drink voluntarily from a 1 mL syringe. This was later used for administration of the NSAID drugs to avoid gavaging the rats. During the study, animal welfare was evaluated by the animal caregivers and veterinarians using a scoresheet developed in-house. The animals were scored twice daily until day 5 post-operatively, daily until day 7, and then twice weekly until day 28. Evaluated parameters included behavior, respiration, external appearance, excretion, body weight, weight-bearing of the operated leg, and wound healing. All rats were weighed pre-operatively, after 3 and 7 days, and weekly afterward. The animals were scanned by micro-CT immediately following surgery (day 0—to confirm appropriate positioning of a screw) and at four further time points post-operatively (see details below). Blood samples were collected from the lateral tail vein pre-operatively and on days 6, 20, and 28 prior to micro-CT scans. On day 28, animals were euthanized with an overdose of pentobarbital via intracardiac injection under sevoflurane anesthesia.

### 4.6. Surgery and Anesthesia

The mean weight (±S.D.) of the rats at surgery was 334 ± 29 g. Anesthesia was induced and maintained with sevoflurane in oxygen. After pre-operative analgesia (buprenorphine 0.03 mg/animal, s.c. Bupaq^®^ ad us. vet., injection solution; Streuli Pharma AG, Uznach, Switzerland) and fluid administration (2 mL pre-warmed Ringer’s solution s.c.), the right tibia was aseptically prepared. Body temperature was maintained using a heating pad with a feedback system (RightTemp, Kent Scientific, Torrington, CT, USA). After the rat was placed in dorsal recumbency, a 1 cm incision was made on the proximomedial aspect of the right tibia. A ø1.2 mm unicortical hole was drilled 2 ± 1 mm distal to the growth plate, then tapped (1.4 mm outer/1.2 mm inner ø). After the pre-inoculated screw was inserted manually, the fascia, subcutis, and skin were closed in three layers using absorbable suture material (Monocryl and Vicryl rapid, Ethicon Inc., Cincinnati, OH, USA; sizes 6–0 and 5–0, respectively). For post-operative analgesia, Tramadol was added to the drinking water (0.5 mg/mL water, Tramal Tropfen 100 mg/mL, Grünenthal, Aachen, Germany) for five days.

### 4.7. Drug Administration

Animals received NSAID treatments orally twice daily over 28 or 7 days, according to their group allocation (ASA: 50 mg/kg, celecoxib: 10 mg/kg, ibuprofen: 40 mg/kg per dose). The dosages were defined by using allometric extrapolation from commonly used dosages in humans [39]. NSAIDs were administered in diluted condensed milk before each treatment and voluntarily taken by the animals from a 1 mL syringe. Each cage contained rats of several groups to avoid cage effects. Additionally, all animals were treated with a combination antibiotic regimen (25 mg/kg rifampicin plus 30 mg/kg cefazolin, s.c.) twice daily from day seven after screw implantation for 14 days, followed by a 7-day washout period to prevent false negative results in the quantitative bacteriological analysis. The seven days prior to the commencement of antibiotic treatment were necessary to allow the infection to develop in the bone.

### 4.8. In Vivo Micro-CT Analysis

The rats were scanned by micro-CT (VivaCT40, Scanco Medical AG, Bruettisellen, Switzerland) immediately post-operatively (day 0), at days 6, 14, 20 under general anesthesia, and after euthanasia (day 28) using a previously established protocol [40]. The operated tibia was pulled to full extension of the stifle joint, and the ankle was fixated in a position of approximately 90° flexion. The scanned region of 10 mm in length and ø25.6 mm field of view was centered, aiming around the implanted screw. The scanner was operated at 70 kV tension, 114 mA current, 220 ms integration time, and 500 projections/180° according to a previously described protocol [40]. The total scanning time was 21 min, and total anesthesia duration was about 30 min per scan. Each micro-CT dataset consisted of 420 slices reconstructed across an image matrix size of 1024 × 1024 pixels, with an isotropic voxel size of 25 μm. Following segmentation of the micro-CT images with a global threshold, bone-implant contact (BIC) and bone fraction (BV/TV) were computed and expressed as percentages of the results of day 0. Bone formation (BF) and bone resorption (BR) were calculated from the rate of temporal changes in BIC and BV/TV. Additionally, periosteal volume and thickness were evaluated. All parameters were computed using automated scripts implemented in the scanner’s software (Image Processing Language, IPL, Scanco Medical).

### 4.9. Total Bacteriology Quantification

After euthanasia, the bones and the screws were independently dissected and collected into sterile containers. Any soft fibrous tissue overlying the screw head was also collected in a separate container. After determining the weight of bone and soft tissue, 1 mL of sterile PBS was added to all the samples. Bacteria adhering to the contaminated screws were determined by sonicating the screws for 3 min, vortexing for 10 s, and ultimately performing serial dilution plating on 5% blood agar plates. The entire tibia from each animal was mechanically homogenized (Omni Tissue Homogenizer and Hard Tissue Homogenizing tips; Omni International, Kennesaw, GA, USA), and the bacteria were quantified by serial dilution on 5% blood agar plates. Soft tissue samples were processed in the same manner. The plates were subsequentially incubated for 24 h at 37 °C and checked for contamination or signs of co-infection.

### 4.10. Histological Processing

Fifteen randomly chosen animals (three for each group) were allocated for histological analysis. Following euthanasia, the lower limb was dissected, and the overlying skin was removed. Afterward, all samples were fixed for at least two weeks in 70% ethanol. After fixation, samples were dehydrated through an ascending ethanol series (70%, 96%, absolute ethanol), with two changes for each step, every 25 days. Then, samples were transferred to xylene and finally to methyl methacrylate (MMA) (Sigma-Aldrich, Buchs, Switzerland) for embedding. The polymerized samples were sectioned using a Leica 1600 annular blade saw (Leica Biosystems Nussloch GmbH, Nußloch, Germany). At least one section in the frontal plane through the center of the screw was made from each sample for Giemsa–Eosin (G–E) staining.

### 4.11. Histomorphometric Analysis (Quantitative)

A quantitative histomorphometric analysis was performed to assess the level of integration of the implant. The open-source ImageJ software (Version 1.53t; LOCI, University of Wisconsin) was used to measure the bone area in a sleeve of 400 µm surrounding the threaded portion of the PEEK screw on each image. The calibration used was 0.602 pixels/µm. The ROI was defined by selecting an identical ROI in the image of the first control sample animal, and this same ROI was applied to all other samples. A polygon was created around the implant to define the ROI. The contrast of the selected images was enhanced to 1% for better visualization. The color threshold channel was utilized to identify the presence of bone structure in the ROI, and the bone area was subsequently measured using the software.

### 4.12. Cytokine Measurements

For each animal, blood samples were collected on days 0 (pre-operative), 6, 20, and 28. Plasma was isolated following centrifugation (400× *g*, 5 min) and stored at −20 °C until analysis for inflammatory cytokines using a V-PLEX Proinflammatory Panel 2 rat kit (Meso Scale Discovery, Rockville, MD, USA) according to the manufacturer’s instructions. Plasma (25 µL) was used for the analysis of the following cytokines: IFN-γ, IL-1β, IL-4, IL-5, IL-6, IL-10, IL-13, KC/GRO, TNF-α.

### 4.13. Statistical Analysis

For the in vitro data, optical density and CFU are shown as mean ± standard deviation (SD). One-way ANOVA with Tukey’s post hoc test for multiple comparisons was used to compare significance between the treatment groups. In the in vivo data, µCT time-series curves are shown as mean ± standard error of the mean (SEM) for the measured data points. Two-way ANOVA with Tukey’s post hoc test for multiple comparisons was used to analyze changes in bone parameters over time and cytokine measurements, and Kruskal–Wallis tests were used to analyze quantitative bacteriology data. Fisher’s exact test was used to check for differences in the proportions of infected animals between groups. The threshold for statistical significance was set as *p* < 0.05. All analyses were performed using GraphPad Prism software, version 9.3.1 (GraphPad Software, Inc., San Diego, CA, USA).

## 5. Conclusions

Our study demonstrated the effects of celecoxib—a selective COX-2 inhibitor—on bone changes and antibiotic efficacy in an *S. epidermidis*-infected rat model. We were able to show that short-term use of celecoxib offered the best option for minimizing bone destruction while also not interfering with antibiotic efficacy compared to the control and long-term NSAID groups. Bone formation was seemingly promoted in the early infection with the use of short-term celecoxib. Further research is needed to elucidate the mechanism underlying the bone protective effect of celecoxib in *S. epidermidis* osteomyelitis and to optimize the use of NSAIDs in fracture patients with bacterial infections. Given the use of NSAIDs as part of multimodal analgesia, and considering the findings from our experimental study, a prospective clinical study is needed to determine the reproducibility of the results in the clinical setting.

## Data Availability

Data are available from the corresponding author on reasonable request.

## Supplementary Materials

The following supporting information can be downloaded at https://www.mdpi.com/article/10.3390/antibiotics13080715/s1, Table S1: overview of the different treatment groups including duration of NSAID and antibiotic treatment. Figure S1: in vitro antimicrobial activity of antibiotics (cefazolin/rifampicin) in combination with other NSAID classes (aspirin/ibuprofen); Figure S2: Quantitative bacteriological assessments after 28 days (all NSAID classes); Figure S3: Bone changes after 28 days (all NSAID classes); Figure S4: Bone change differences over the 28 days of long- and short-term celecoxib treatment in comparison to the control group. Figure S5: cytokines following short- and long-term celecoxib treatment; Figure S6: cytokines following long-term treatment with other NSAIDs (aspirin/ibuprofen).
