# Supplementary material for: Short-Term Celecoxib Promotes Bone Formation without Compromising Cefazolin Efficacy in an Early Orthopaedic Device-Related Infection: Evidence from a Rat Model
Source: Antibiotics (Basel). 2024 Jul 30;13(8):715. doi: 10.3390/antibiotics13080715 (PMC11350844; doi:10.3390/antibiotics13080715)
Supplement: Supplementary file 1 [file antibiotics-13-00715-s001.zip › antibiotics-3109470-supplementary.pdf]

Short-term Celecoxib Promotes Bone Formation Without Compromising Cefazolin Efficacy in Early Orthopaedic Device-Related Infection: Evidence from a Rat Model

Vuyisa Siphelele Mdingi[1,2], Lena Gens[1], Karen Mys[1], Peter Varga[1], Stephan Zeiter[1], Leonard Charles Marais[2], Geoff Robert Richards[1], Fintan Thomas Moriarty[1] and Marco Chittò[1,*]

[1]AO Research Institute Davos, Davos, Switzerland

[2]Department of Orthopaedic Surgery, School of Clinical Medicine, University of KwaZulu Natal, South Africa

[*]Correspondence: marco.chitto@aofoundation.org

Supplementary Materials

NSAID groups	**Antibiotic**	**NSAID treatment**	**Antibiotic Treatment**	**Number of animals**
/	Rifampin / Cefazolin	Day 0 to Day 28	Day 7 to Day 14	15
Aspirin	Rifampin / Cefazolin	Day 0 to Day 28	Day 7 to Day 14	12
Ibuprofen	Rifampin / Cefazolin	Day 0 to Day 28	Day 7 to Day 14	12
Celecoxib	Rifampin / Cefazolin	Day 0 to Day 28	Day 7 to Day 14	12
Celecoxib (S)	Rifampin / Cefazolin	Day 0 to Day 6	Day 7 to Day 14	12

Supplementary Table S 1: overviewing of the different treatment groups including duration of NSAID and antibiotic application as well as the number of animals allocated per each group.

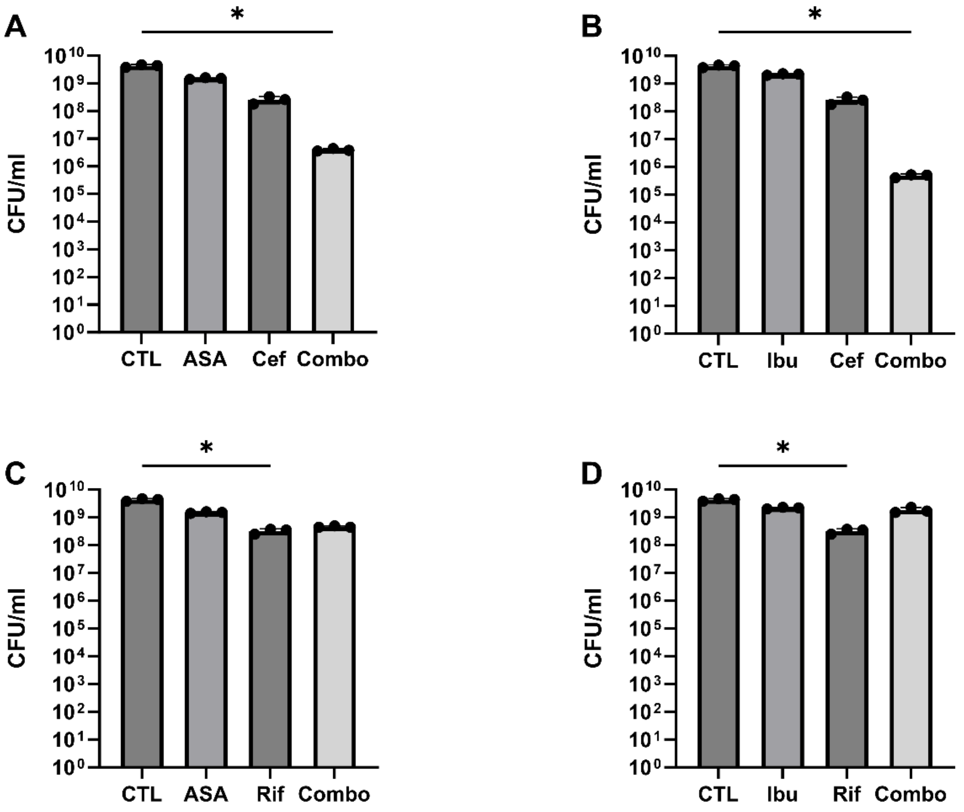

Supplementary Figure S 1 shows the antimicrobial interaction of cefazolin (Cef) and NSAIDs, acetylsalicylic acid (ASA) or ibuprofen (Ibu) alone or in combination (A & B) and rifampicin (Rif) and NSAIDs alone or in combination (C & D). The combined treatment of Cef and Asp or Ibu resulted in a two- and three-log reduction respectively when compared to the Cef only. Conversely, the single treatment of Rif resulted in a one-log reduction if compared to the dual drug application mode. CFU data are shown. Each bar plot represents the average of three independent experiments with three replicates per experiment. A one-way ANOVA with Tukey's *post*-hoc test was performed to determine significance between the groups. The standard deviation and significance level are shown * $p<0.05$.

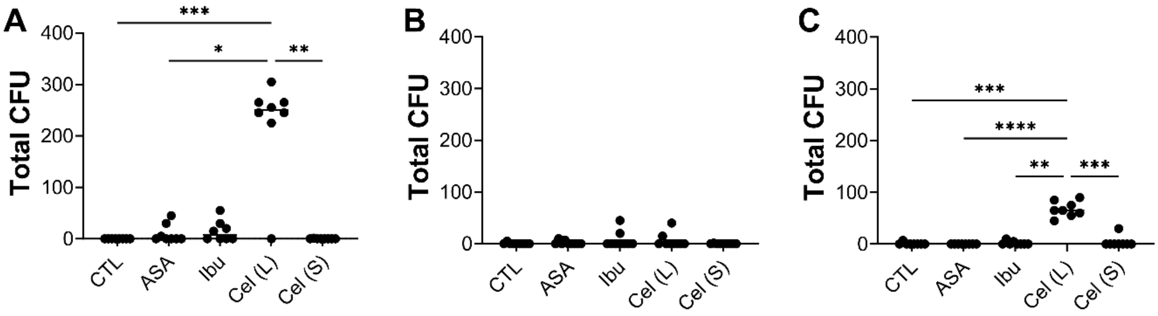

Supplementary Figure S 2. Quantitative bacteriological assessments after 28 days of the control (CTL), acetylsalicylic acid (ASA), ibuprofen (Ibu), and long (Cel(L)) and short (Cel(S)) celecoxib groups. Data shown are the total CFU counts from each animal from bone (A), soft tissue (B) and screw (C) with the mean and the standard deviation. A Kruskal-Walli's test with Dunn's *post*-hoc test for multiple comparisons was performed to compare differences in CFU counts between groups. The significance levels are shown * $p<0.05$, ** $p<0.01$, *** $p<0.001$, **** $p<0.0001$.

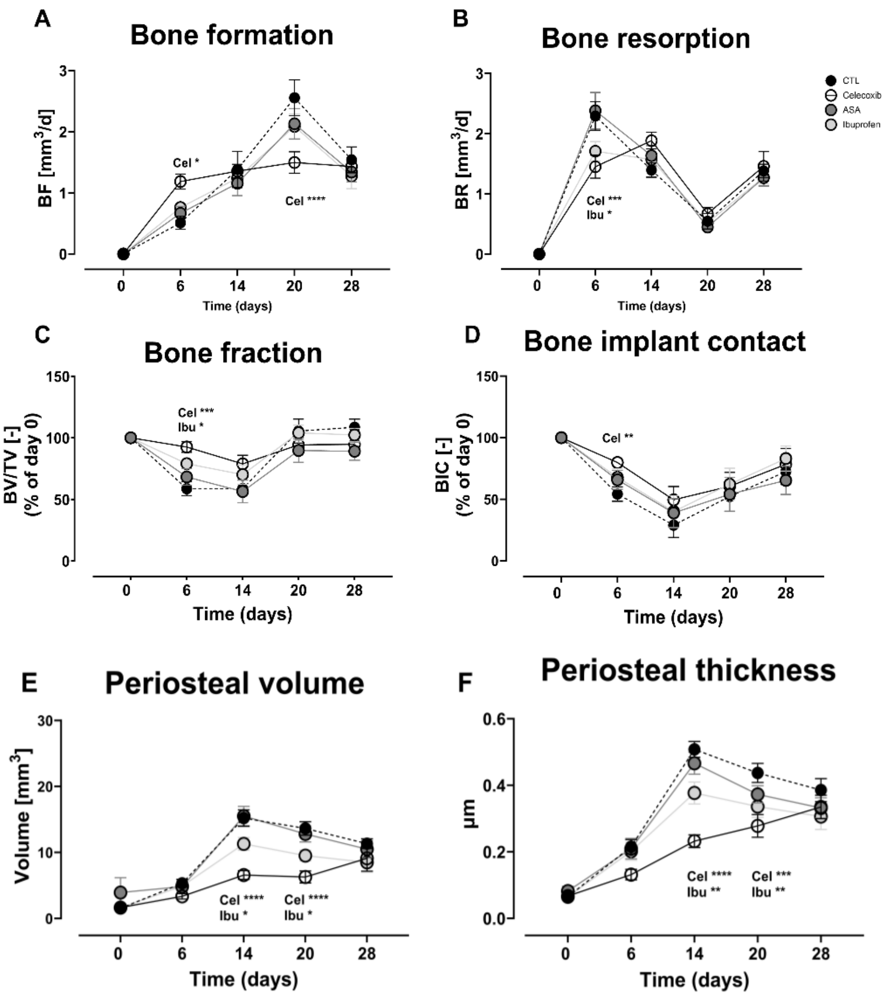

Supplementary Figure S 3 differences in (A) bone formation and (B) bone resorption, (C) bone fraction and (D) bone implant contact, and (E) periosteal volume and (F) periosteal thickness over time during different NSAID treatments with long celecoxib (Cel(L)), acetylsalicylic acid (ASA) and ibuprofen (Ibu) treatment of an *S. epidermidis* infection. Data shown are the median ± SEM. A two-way ANOVA with Dunnett's *post-hoc* test was performed to determine significant differences between treatment groups and the control group. * p<0.05, ** p<0.01, *** p<0.001, **** p<0.0001.

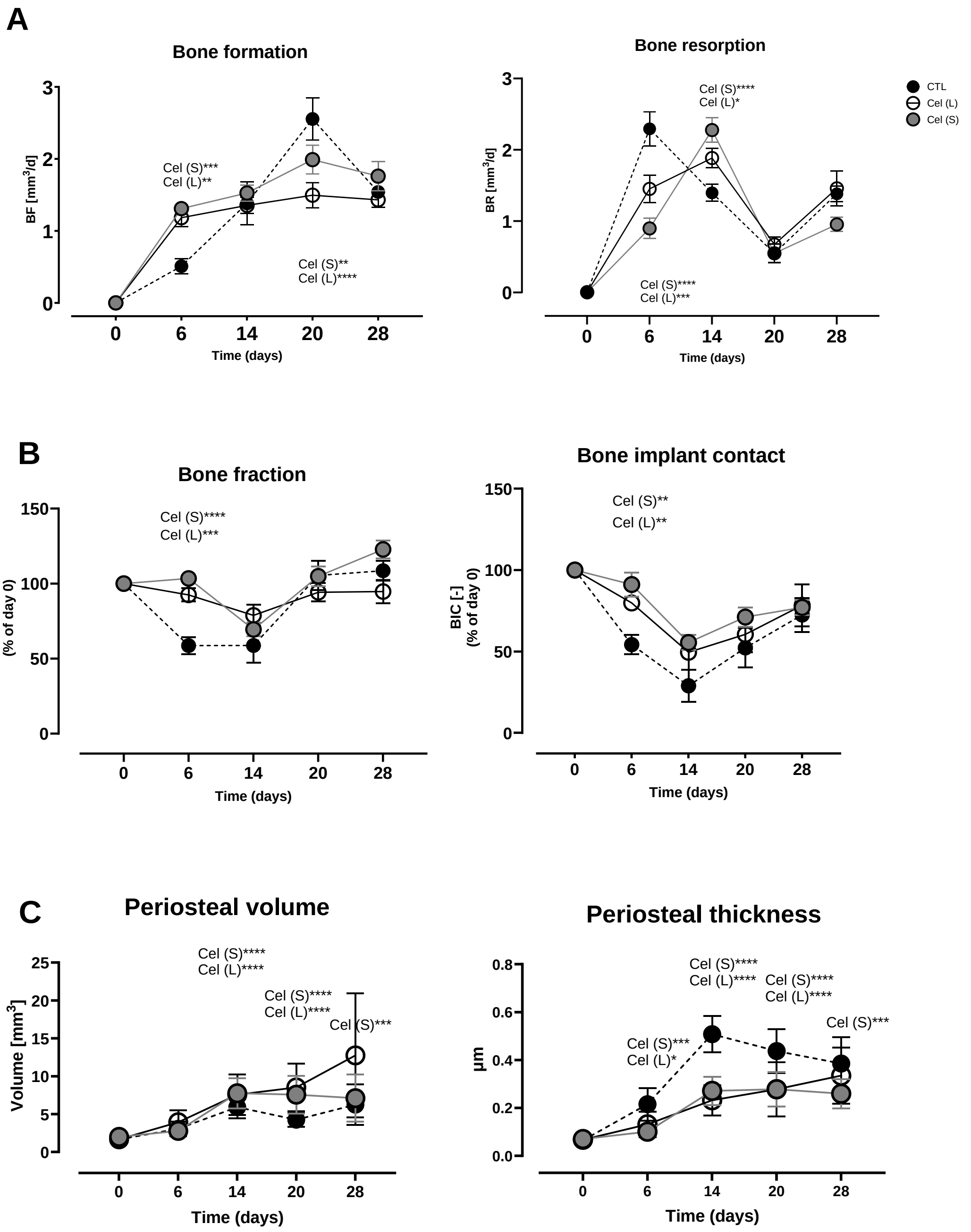

Supplementary Figure S 4 differences in (A) bone formation and (B) bone resorption, (C) bone fraction and (D) bone implant contact, and (E) periosteal volume and (F) periosteal thickness over time during long celecoxib (Cel(L)), short celecoxib (Cel(S)) treatment of an *S. epidermidis* infection compared to the control group. Data shown are the median ± SEM. A two-way ANOVA with Dunnett's *post-hoc* test was performed to determine significant differences between treatment groups and the control group. * $p<0.05$, ** $p<0.01$, *** $p<0.001$, **** $p<0.0001$.

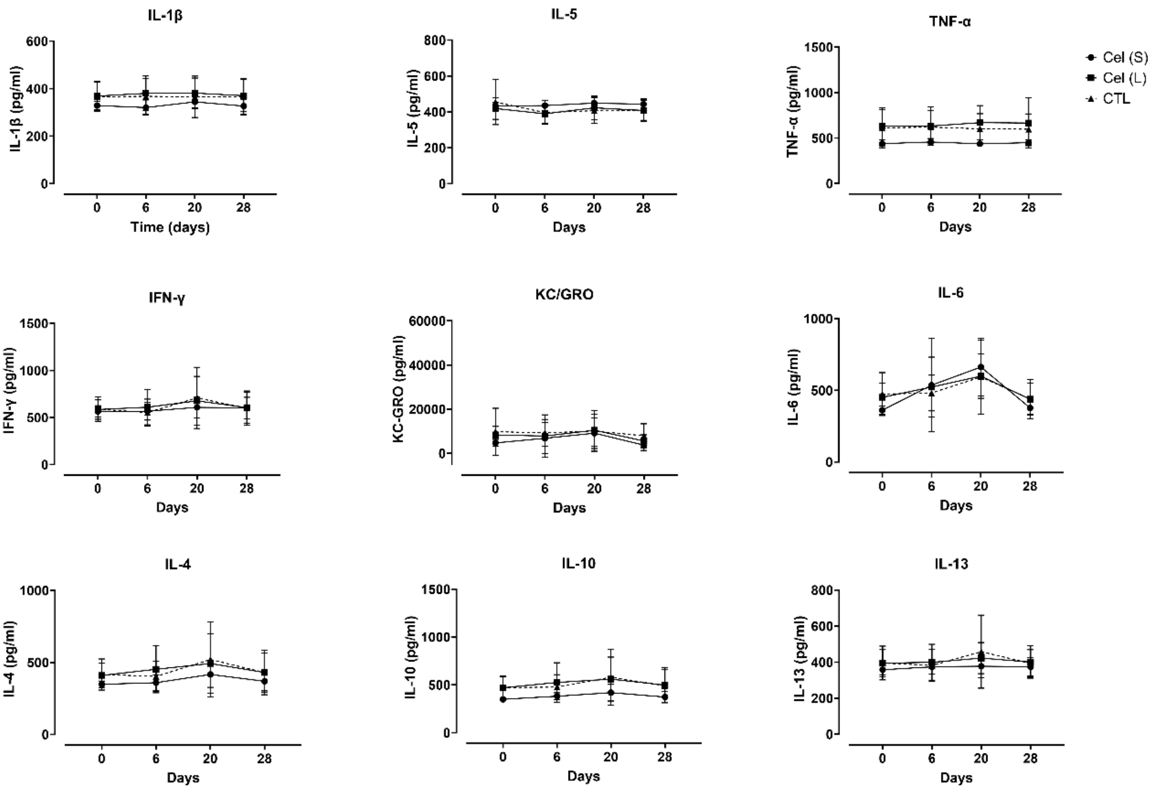

Supplementary Figure S 5 Cytokines secretion after the influence of short Cel (S) and long Cel(L) celecoxib treatment compared to the control (CTL) group. Blood samples collected during the study (day 0, 6, 20 and 28) were processed for inflammatory cytokines secretion analysis using a multiplex immunoassay (n=12). Cel(L) treatment started at day 0 till day 28 while the Cel(S) was administrated from day 0 to day 7. Data shown are the median ± SEM.

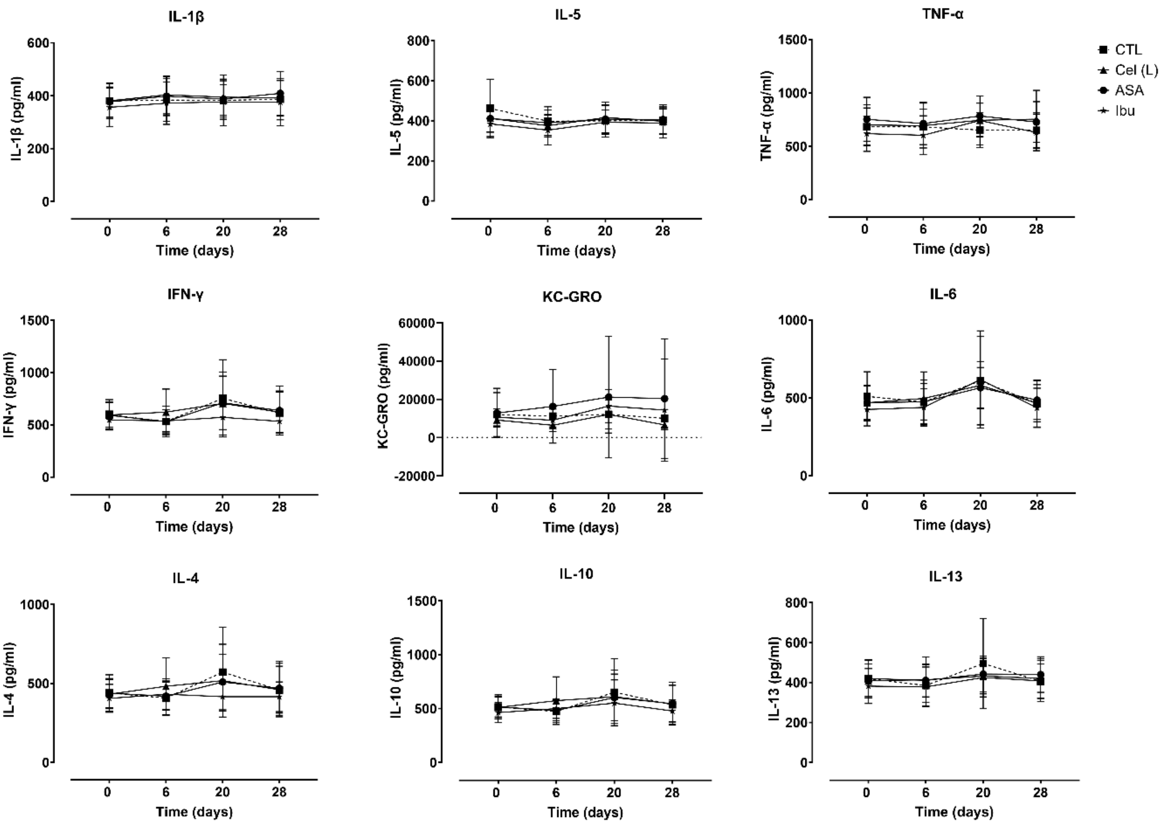

Supplementary Figure S 6 Cytokines secretion after the influence of long-term NSAID administration measured at different time points. Blood samples collected during the study (day 0, 6, 20 and 28) from control (CTL), celecoxib (Cel(L)), acetylsalicylic acid (ASA) and ibuprofen (Ibu) were processed for inflammatory cytokines secretion analysis using a multiplex immunoassay (n=12). Data shown are the median ± SEM.
